# Sex-specific disruption of estrogen receptor alpha (ERα) density in the social brain neural network by Firemaster 550

**DOI:** 10.1210/jendso/bvag050

**Published:** 2026-03-16

**Authors:** Jinyan Cao, Sagi Enicole A Gillera, William P Marinello, Heather B Patisaul

**Affiliations:** Department of Biological Sciences, North Carolina State University, Raleigh, NC 27519, USA; Division of Translational Toxicology (DTT), National Institute of Environmental Health Sciences (NIEHS), Durham, NC 27705, USA; Department of Biological Sciences, North Carolina State University, Raleigh, NC 27519, USA; Department of Biological Sciences, North Carolina State University, Raleigh, NC 27519, USA; Department of Biological Sciences, North Carolina State University, Raleigh, NC 27519, USA; Division of Translational Toxicology (DTT), National Institute of Environmental Health Sciences (NIEHS), Durham, NC 27705, USA

**Keywords:** organophosphate ester, OPE, neurodevelopmental disorder, NDD, endocrine disruptor, EDC

## Abstract

We investigated how developmental exposure to the commercial flame retardant (FR) mixture Firemaster 550 (FM 550) affects estrogen receptor alpha (ERα) expression in key brain regions related to sociosexual behavior. We used prairie voles, a socially monogamous, biparental rodent species of high translational human relevance. This study used adult siblings from a prior behavioral study showing developmental FM 550 exposure impaired a range of socioemotional behaviors in adults including loss of partner preference in males. Dams were exposed to FM 550 (500, 1000, or 2000 µg/day) via subcutaneous injections throughout gestation, and pups were directly exposed from birth to weaning. ERα immunoreactive (ERα-ir) neuron numbers and mRNA expression levels were quantified in subregions of the social brain neural network (SBNN). As anticipated, FM 550 impacts were sex-, dose-, and region-specific, with FM 550 tending to increase ERα-ir cell numbers in the anterior hypothalamus regardless of sex, but decrease them in the female mediobasal hypothalamus, amygdala, and extended amygdala. These studies demonstrate that developmental FR exposure impacts adult SBNN ERα availability and provide support that disrupted ERα action in the SBNN may be a mechanism underlying disruption of socioemotional behavior, energy balance, and related neuroendocrine physiology. Impacted ERα neuronal populations are also influenced by other receptors, neuropeptides, neurosteroids, and signaling molecules to govern prosocial behaviors, which is the ongoing direction of this work. Collectively, these data add to growing evidence that FM 550 FRs are neuroendocrine disruptors that can induce persistent impacts across developing socioemotional pathways and systems.

Epidemiological evidence consistently links developmental exposure to flame retardants (FRs) and other endocrine disrupting chemicals (EDCs) to rapidly rising rates of neurodevelopmental disorders (NDDs), including autism spectrum disorder (ASD), with growing recognition that environmental factors may play a significant if not comparable role to genetics in NDD etiology [1-5]. The mechanisms by which this occurs, however, remain uncertain. Although some brominated FRs have been phased out of use due to their endocrine disrupting properties, emerging new approach methodology (NAM) data and integrated health approaches to testing and assessment have identified organophosphate ester (OPE) and other replacement FRs as EDCs and developmental neurotoxicants (DNTs) [6-10], suggesting they may be regrettable substitutions [11, 12]. Using the prairie vole (*Microtus ochrogaster*), a uniquely suitable animal model of prosocial behaviors, and the Research Domain Criteria (RDoC) Project framework, an experimental schema specifically designed to help elucidate the biological basis of behavioral traits underlying mental health disorders [13, 14], we tested the hypothesis that early life exposure to a commercial FR mixture containing both brominated and OPE components, Firemaster 550 (FM 550), disrupts estrogen receptor alpha (ERα) content in subregions of the social brain neural network (SBNN).

FM 550 comprises 2 brominated flame retardants (BFRs), (2-ethylhexyl-2,3,4,5-tetrabromobenzoate and bis(2-ethylhexyl) 2,3,4,5-tetrabromophthalate), the OPE FR (OPFR) triphenyl phosphate (TPHP), and numerous TPHP analogs with varying degrees of aryl isopropylation (collectively called ITPs) at a roughly 50:50 BFR:OPFR ratio [15-17]. FM 550 components are used in other FR mixtures along with myriad other products, including personal care products, resulting in nearly unavoidable and expanding human exposure [18-21]. Household dust is one of the most common sources with levels of TBB and TBPH, for example, ranging from 300 to 400 ng/g on average, and upwards of 10 000 ng/g in 1 study [22]. Although levels vary, children consistently have higher FR exposures than adults [23-26] (as much as 5× higher according to 1 study [24]), making them particularly vulnerable to their DNT and endocrine disrupting properties [4, 27, 28]. Globally, FM 550 components are consistently detected in breast milk (∼0.1 µg/kg body weight/day [21]), blood, hair, fingernails, urine, and other human tissues [18, 22, 29]. In children, particularly boys in some studies, exposure has been associated with clinically relevant behavioral problems including social behavior problems [30], externalizing problems, cognitive delay, and hyperactivity and attention deficits [31, 32].

We and others have repeatedly identified FM 550 and its component classes as endocrine disrupting and developmentally neurotoxic [6, 11, 12, 22, 33-43] with socioemotional and other behavioral deficits consistently observed in multiple species following developmental exposure [6, 43-48]. Most commonly reported effects include heightened anxiety in females and impaired sociality in both sexes, outcomes concordant with published human and NAM data [6, 12, 31, 43-48]. The present studies build on our related, published work showing perinatal FM 550 exposure at doses that produce internal levels modeling the upper range of childhood and occupational exposure (500, 1000, or 2000 µg/day) impaired a range of NDD-relevant socioemotional behaviors including affiliation and attachment in adult prairie voles [45, 47, 49]. Most strikingly, in all 3 prior studies, partner preference, an indicator of pair bonding, was absent in the FM 550-exposed males regardless of dose, suggesting impaired attachment.

EDCs, a class of substances that interfere with endocrine system organization and function are well known to sex-specifically disrupt neurodevelopment, even at human-relevant exposure levels [50-55]. Disruption of ERα expression and density is a well-characterized mode of EDC action that can alter the organization and function of neuroendocrine systems and, consequently, socioemotional and related behavior [51, 56-58]. Significantly, transcriptomic analysis of the newborn rat cortex following gestational FM 550 exposure identified ERα as a potential “hub protein” driving further downstream effects [59]. The experiments herein measured ERα mRNA expression and immunoreactive neuron numbers in adult animals across the SBNN to help advance understanding of the possible mechanisms by which observed neuroendocrine and socioemotional deficits arise following developmental FM 550 exposure.

The SBNN is the central coordinator of socioemotional behaviors [60-62]. First described in the 1990s [62], it comprises a group of limbic areas, including nuclear groups within the medial (MeA) and extended amygdala (such as the bed nucleus of the stria terminalis [BNST]), the lateral septum (LS), the medial preoptic area (MPOA), the anterior hypothalamus, the ventromedial nucleus (VMN) and adjacent ventrolateral hypothalamus, and the midbrain periaqueductal gray and adjacent tegmentum. Subsequent research has refined and enhanced the model to incorporate additional brain areas including the paraventricular nucleus, cortical (eg, medial prefrontal cortex), subcortical (eg, nucleus accumbens), and brainstem (eg, ventral tegmental area) regions [62-67]. Each area is reciprocally interconnected and sexually dimorphic. ERα is highly expressed throughout the rodent SBNN of both sexes, particularly in hypothalamic and limbic regions [68], and related regions that coordinate reproductive and metabolic functions such as the arcuate nucleus (ARC) and the anteroventral periventricular nucleus (AVPV).

In addition to species and sex differences, there are striking differences in regional ERα expression levels between monogamous and polygynous species including highly related species of *Microtus* and *Peromyscus* [69], highlighting its role in attachment-related traits. Specifically, ERα levels in the MPOA, MeA, and BNST directly contribute to the propensity to spontaneously display prosocial traits, such as pair-bond formation and paternal care; behaviors found in humans but not typical laboratory rats or mice. For example, low levels of ERα in the male BNST and MeA accompany high levels of partner attachment (monogamy), affiliation, and parental care [69]. Other key contributing estrogen-sensitive hormones include oxytocin and vasopressin (AVP) with their receptor density also markedly differing between monogamous and nonmonogamous species, including humans and other primates [70-74].

Hence, for this work, we used prairie voles (*Microtus ochrogaster*), a biparental and monogamous rodent, because they are more translational and human-relevant than traditional laboratory rodent species for the endpoints of interest. Although largely unfamiliar to toxicologists, this animal model has been used for decades in the neurosciences to explore the biological basis of attachment, social cognition, affiliation, and communication [75, 76]. They have also been used to probe the etiology of NDDs with social deficits including ASD and potential therapeutics [64, 72, 77, 78]. While there is no, and can never be, animal model for a uniquely human condition like ASD, hallmark traits such as impaired attachment can be reliably modeled [73].

Finally, the RDoC, spearheaded by the National Institute of Mental Health, provides an experimental framework for linking NDD-relevant behavioral traits to their underlying biological basis at various levels including circuits, cells, and genomes [13, 14]. Thus, we assert it constitutes a powerful tool for mechanistically linking chemical exposures to NDDs including those with social deficits like ASD. The RDoC supports the hypothesis that ERα expression in SBNN nuclei could be a mode of action by which developmental FM 550 exposure sex-specifically impairs male attachment and elevates female anxiety-like behaviors. By leveraging a uniquely suitable animal model with high translational relevance [73, 79] in combination with the RDoC framework, the present studies provide a powerful alternative to traditional guideline toxicology studies (all of which lack assessments of socioemotional behavioral deficits or their neural underpinnings) to achieve a more “fit for purpose” in vivo approach while adhering to the “3Rs.” Hence, the present studies advance our mechanistic understanding of how FM 550 disrupts neuroendocrine systems fundamental to healthy socioemotional regulation.

## Materials and methods

### Rationale and prior, related studies

The tissues used herein came from the same animals in a previously published study examining different neural endpoints [49]. Those animals were siblings of prairie voles used in a prior behavioral study revealing socioemotional impairments following developmental FM 550 exposure [47]. Hence, the tissues used for the present study came from animals that underwent the same exposure paradigm as the related, 2 prior ones. Here we quantified ERα expression in the SBNN plus related ERα-rich regions that interface with it: AVPV, MPOA, ARC, VMN, MeA, BNST, and the ventral part of the LS (LSV). All contain neurons expressing gonadal hormone receptors, either ERα, Erβ, or G protein-coupled estrogen receptor 1 (GPER), are sexually dimorphic, and sensitive to endogenous estrogen and EDCs [61, 68, 80-85]. We only focused on ERα because available tissue was limited and reliable antibodies for the other 2 were not available. Because nearly all psychological conditions including ASD, attention deficit disorder and anxiety disorders, have a sex-biased prevalence, and wide phenotypic heterogeneity, particularly between boys and girls [86, 87], sex-specific outcomes were accounted for and anticipated.

### Animal care and maintenance

Animal care, maintenance, and experimental protocols met the standards of the Animal Welfare Act and the U.S. Department of Health and Human Services “Guide for the Care and Use of Laboratory Animals’ and were approved by the North Carolina State University (NC State) Institutional Animal Care and Use Committee. A supervising veterinarian approved and monitored all procedures throughout the duration of the project. Data reporting adheres to the ARRIVE (Animal Research: Reporting of In Vivo Experiments) guidelines “Essential 10.” These guidelines were developed in consultation with the scientific community as part of an NC3Rs (National Centre for the Replacement Refinement and Reduction of Animals in Research) initiative to improve the standard of reporting of research using animals.

The NC State prairie vole (*Microtus ochrogaster*) colony was derived from founders generously gifted by Bruce S. Cushing at the University of Texas El Paso in 2017 and bred in-house in a manner that maintains genetic diversity in humidity- and temperature- controlled rooms at 22 °C and 30% average humidity, each with 12 hour:12 hour light:dark cycles (lights on at 6 Am EST) in the Association for Assessment and Accreditation of Laboratory Animal Care-approved Biological Resource Facility at NC State. Food (Lab Diet 5326 high-fiber rabbit diet) and water were provided ad libitum. As in our prior studies, and in accordance with recommended practices for EDC research [88], all animals were housed in conditions specifically designed to minimize unintended EDC exposure, including use of reverse osmosis water, glass water bottles with metal sippers, woodchip bedding, and thoroughly washed polysulfone caging. Normally, we would use a phytoestrogen-free diet, but high fiber and at least some phytoestrogen content is required to maximize health and fertility of this herbivorous, diurnal, species (National Research Council (U.S.). Subcommittee on Laboratory Animal Nutrition., 1995).

### FM 550 preparation and exposure

All dosing solutions were prepared as previously described [36, 42, 47] in Heather Stapleton's laboratory at Duke University and transferred to the Patisaul laboratory at NC State. In brief, the FM 550 commercial mixture was obtained from Great Lakes Chemical (West Lafayette, IN, USA) [89] and each dose (0, 500, 1000 and 2000 μg/20 μL vehicle) prepared by diluting an appropriate amount of the concentrated solution in sesame oil (Sigma), stirred for 6 hours, then stored in amber bottles at 4 °C until use. FM 550 doses were selected based on our prior neurobehavioral work in rats and below the purported NOAEL of 50 mg/kg/day for the brominated FRs in the mixture (3790).

Dosing was based on the average mid-gestationally pregnant female body weight (BW) in the colony (50 g) rather than each individual BW to avoid the stress of repeated handling during exposure. Thus, the dams were effectively dosed at approximately 0 mg/kg BW, 10 mg/kg BW, 20 mg/kg BW, and 40 mg/kg BW. Because voles breed continuously and generally become pregnant 48 hours after giving birth, a subset of dams generated from the breeding colony were injected on the day after parturition, designated as gestational day 0. Dams were randomly assigned to an exposure group using a random number generator and were subcutaneously (s.c.) injected with FM 550 (0, 500, 1000, 2000 μg) from gestational day 0 through the day of the experimental offspring birth. No dosing occurred on the day of birth. Offspring were then dosed directly by s.c. injection from the day after parturition, postnatal day (PND) 1, until weaning, PND 21. Dosing occurred daily beginning at 14:00 hours for all animals.

Although oral exposure is considered more human relevant, s.c. injection was chosen because the pharmacokinetics of FM 550 in prairie voles was unknown at the time and not assumed to be similar to other rodents given that prairie voles are herbivorous, and their kidneys evolutionarily specialized to scavenge water. Our previous publication using the same exposure paradigm assessed internal dose [47], in a subset of sexually naïve adult voles (4 males and 5 females) exposed to FM 550 (2000 µg/day) for 5 days via s.c. injection with blood collected 4 hours after injection. Serum levels of 2-ethylhexyl-2,3,4,5-tetrabromobenzoate were the most abundant between 15 and 25 ng/mL, and TPHP and several of the ITP isomers ranged from 0.2 to 20 ng/mL. BEH-TEBP accounts for ∼14% of FM 550 [34], but was not detected in the serum, likely because of its rapid clearance [90]. Several ITP isomers were detected in female but not male serum. Fetal levels are unknown.

The 4 exposure groups were designated as follows: 0 µg FM 550 (CONTROL), 500 µg FM 550 (LOW), 1000 µg FM 550 (MED), and 2000 µg FM 550 (HIGH). Litter was the statistical unit and sample size (labeled in each table and graph) varied across group and region of interest (ROI) due to the availability of suitable sections, section quality, and staining quality. Specific details about what was included and excluded for each endpoint is provided in the results and Table 1.

**Table 1:** Number of sections averaged for each ROI for IHC ERα-ir cell counting and mRNA expression quantification

ROI (number of sections averaged)	Exposure	FM 550-0 µg CONTROL		FM 550-500 µg LOW		FM 550-1000 µg MED		FM 550-2000 µg HIGH		RNAScope	0 µg CONTROL		2000 µg HIGH	
	Sex	N	Sections # (n)	N	Sections # (n)	N	Sections # (n)	N	Sections # (n)	ROI (section # for average)	N	Sections # (n)	N	Sections # (n)
**AVPV (3 sections)**	F	10	2(1)	13		10		8	2(1)	**AVPV**	6		9	
	M	14		10		12		9		**(2 sections)**	11		10	
**MPOA (3 sections)**	F	10	2(1)	12		10	2(1)	8	2(1)	**MPOA**	5		10	
	M	14		10		10		12	2(1)	**(2 sections)**	8		10	
**ARC (4 sections)**	F	8		12		10		8				N/A		
	M	12		10		10		10						
**VMNvl (4 sections)**	F	8		9		9	3(1)	10	3(1), 1(1)					
	M	13		9	3(1), 1(1)	8		10						
**BNSTa (3 sections)**	F	8	2(1)	11		10		8		**BNSTa**	7		9	
	M	14		11		11		10		**(4 sections)**	10		10	
**BNSTp (3 sections)**	F	9	2(1)	10	2(1)	11	2(1)	8	2(1)	**BNSTpm**	6	1(1)	10	
	M	14		9		8	2(2)	12	2(2), 1(1)	**(2 sections)**	7		10	
**MePD (4 sections)**	F	5		11	3(1), 2(1)	10	3(2), 2(3)	8	3(1)	**BNSTpl**	6	1(1)	10	
	M	12	3(1)	9	3(1)	8		9		**(2 sections)**	7		10	
**LSV**	F	7		10		9		8		**LSV**	3		8	
**(2 sections)**	M	11	1(1)	10		7		9	1(3)	**(2 sections)**	12		12	

The first column indicates the number of sections used for each ROI to quantify ERα-ir, while the first column in the bolded area indicates the number of sections used for each ROI to quantity ERα mRNA expression. N = sample size (number of animals) in each group. For some animals the target number of samples were not available. The number of instances that occurred for each ROI plus the number of sections available are depicted in the Sections # (n) columns where the number of sections used is (n).

Abbreviations: ARC, arcuate nucleus; AVPV, anteroventral periventricular nucleus; BNSTa, bed nucleus of the stria terminalis, anterior part; BNSTp, bed nucleus of the stria terminalis, posterior part; BNSTpl, bed nucleus of the stria terminalis, lateral part; BNSTpm, bed nucleus of the stria terminalis, posterior medial part; ERα-ir, estrogen receptor α immunoreactive; F, female; FM 550, Firemaster 550; LSV, ventral part of the lateral septum; M, male; MPOA, medial preoptic area; N/A, data not available; ROI, region of interest; VMNvl, ventromedial hypothalamic nucleus ventrolateral part.

### Tissue collection

Animals were humanely euthanized by transcardial perfusion, and the brains removed, cryoprotected, and flash frozen as previously described for the related, prior study [49] on PNDs 77-85. Estrous cycle did not need to be standardized at sacrifice because prairie voles are induced ovulators. Brains were sectioned at 40 µm with a freezing sliding microtome (Leica 1850). Four serial sets of sections were collected, with some sections used for a prior, related study [49]. From the remaining tissue collection, sections containing the ROIs of interest were selected and processed for either in situ histochemistry hybridization or immunohistochemistry (IHC).

### In situ histochemistry hybridization

**Figure 1:**
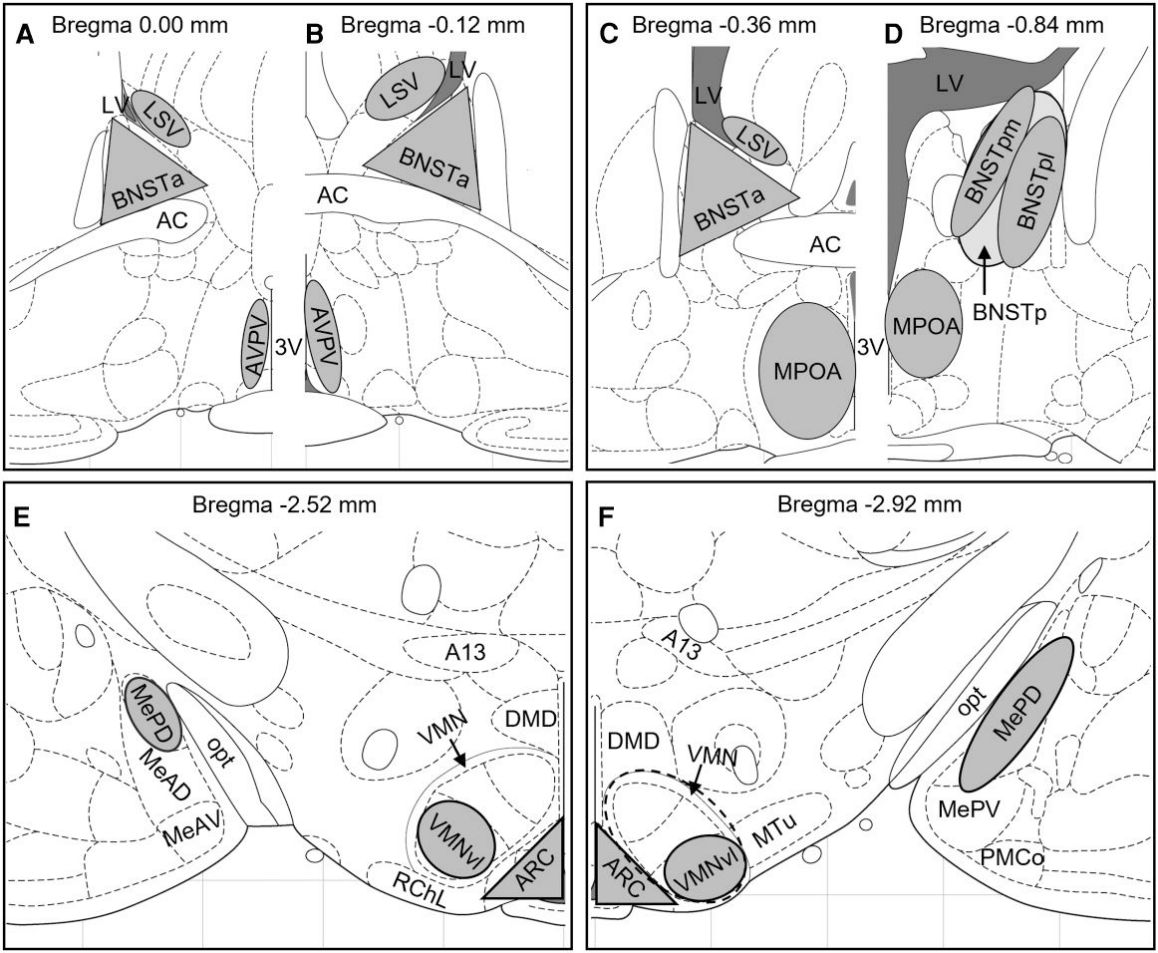
For each target brain area, a region of interest (ROI) template was created with the assistance of a standard rat brain atlas (Paxinos and Watson 2007), which are depicted here. ROIs included AVPV (A and B), MPOA (C and D), BNSTa (A to C), BNSTp (both BNSTpm and BNSTpl, (D), ARC and VMNvl (E and F), and MePD (F).

In situ histochemistry hybridization was used to quantify ERα mRNA expression in the following ROIs: AVPV, MPOA, BNST, and LSV (Fig. 1). In situ histochemistry hybridization was performed using RNAScope technology (Advanced Cell Diagnostics, Newark, CA) with probes specifically designed for prairie vole. Pilot labeling in test tissue revealed background of RNAScope-labeled sections was relatively high, likely because the tissue was fixed which is not optimal for RNA quantification. Consequently, RNAScope was only performed on the CONTROL and HIGH groups to conserve tissues and reagents, and for the specific purpose of determining how concordant protein and mRNA expression were across regions and experimental groups. All sections were first washed multiple times with 1× PBS, slide-mounted (Fisher Superfrost Plus, catalog number 12-550-15), and dried overnight at room temperature (RT) with a protective cover. in situ histochemistry hybridization was then performed according to the manufacturer's instructions by first postfixing the dried mounted sections in 10% neutral buffered formalin at 4 °C for 30 minutes, then rinsed in 1× PBS, and dehydrated in ascending concentrations of ethanol (50%, 70%, 100%). The slides were then dried completely and boundaries drawn around the sections with a hydrophobic pen (ImmEdge PAP pen; Vector Labs). Once dry, all slides were then placed in slide trays designed specifically for RNAScope and a series of pretreatments performed per the manufacturer's instructions. Slides were first incubated with hydrogen peroxide for 10 minutes at RT, then washed in ddH_2_O. All slides were then submerged in RNAScope target retrieval reagent solution for 10 minutes at 100 °C, then immediately transferred into ddH_2_O and washed 3 times. Slides were dried completely at RT after rinsing in 100% ethanol and a protease III incubation was then done at 40 °C for 30 minutes. Following 1× PBS washes, all slides were incubated with an appropriate amount of probe mix containing RNAScope Probe-Mo-Esr1-C1 (ACD) according to the user manual. Then all slide trays with wet humidifying paper were placed in a HybEZ oven (ACD) for 2 hours at 40 °C. ERα signal was amplified and developed with RNAScope multiplex fluorescent v2 Assay Kit (ACD). All slides were washed 2 times in 1× RNAScope wash buffer (diluted from 50×) and returned to the 40 °C oven for 30 minutes after submersion in AMP1 amplification reagent. Washes and amplification were repeated using AMP 2 and AMP 3 with a 30-minute and 15-minute incubation period, respectively. Following amplification, ERα signal was developed at 40 °C with HRP-C1 solution for 15 minutes, with Opal 570/TSA (AKOYA Bioscience) for 30 minutes, and HRP blocker for 15 minutes sequentially with wash buffer rinsing after each step. Finally, the wash buffer was gently flicked off and the slides were cover slipped with ProLong Gold Antifade Mountant (Invitrogen). The covered slides were stored in the dark at 2 through 8 °C until confocal scanning.

### Immunohistochemistry

Immunohistochemistry proceeded using longstanding protocols in our laboratory [91] and validated antibodies. Sections (4-8 per animal depending on availability) containing each ROI were placed into 1× potassium phosphate buffer solution (0.02 M KPBS). All sections were washed in 1× cold KPBS and preincubated in 1× KPBS with 0.3% Triton X-100 and 2% normal goat serum for 24 hours at 4 °C. Sections were then incubated in a primary antibody cocktail consisting of 1× KPBS with 1:6000 rabbit polyclonal anti-ERα (sc-542 Santa Cruz; RRID: AB_631470 generously gifted by Dr. Scott Belcher, NC State University) on a shaker at 4 °C. After 72 hours’ incubation, sections were washed and incubated with Alexa Fluor 555 goat anti-rabbit (Invitrogen; A21428 [1:200]) secondary antibody in normal goat serum for 90 minutes. After a final series of washes in 1× KPBS, sections were mounted on Fisher Superfrost Plus glass slides (catalog number 12-550-15), coverslipped with a glycerol mountant, and stored at −20 °C until imaging.

### ROI identification, imaging, and image analysis

As is commonly done for prairie vole neuroanatomy [92, 93], the Paxinos and Watson rat brain atlas (Paxinos and Watson 2007) was used as a reference to identify the nuclei of interest. The borders of each ROI were then bounded for quantification using predefined shapes as shown in Fig. 1. Confocal microscopy was used to image ERα immunoreactive (ERα-ir) cells and fluorescently tagged ERα mRNA transcripts (Fig. 1). ROIs included the AVPV (Fig. 1A and 1B), from Bregma 0.00 to −0.12 mm; MPOA (Fig. 1C) from Bregma −0.24 to −0.72 mm; BNST medial division, from bregma 0.00 to −0.96 mm; and LSV part (Fig. 1A to 1C) from Bregma 0.36 to −0.12 mm; arcuate (ARC, Fig. 1E and 1F), from Bregma −2.04 to −3.12 mm; ventromedial hypothalamic nucleus ventrolateral part (VMNvl, Fig. 1E and 1F), from Bregma −2.04 to −3.12 mm; MeA, posterodorsal part (MePD, Fig. 1F), from Bregma −2.76 to −3.12 mm). The BNST is a complex, oblong region with multiple subnuclei. Here, we quantified ERα signal in a region we collectively labeled the medial division of the BNST, anterior part (BNSTa), from Bregma 0.00 to −0.48 mm (Fig. 1A to 1C), and medial division of the BNST, posterior part (BNSTp), from Bregma −0.72 to −0.96 mm (Fig. 1D). In the RNAScope labeled sections, we were also able to identify subdivisions of the BNSTp including the BNSTp medial part (BNSTpm) and the lateral part (BNSTpl).

All ROIs examined are well known to express steroid hormone receptors and are highly sexually dimorphic nuclei [94-96]. The AVPV and ARC are essential for reproductive physiology and behavior, most significantly ovulation in females, whereas the MPOA, BNST, LSV, amygdala, and VMNvl belong to the SBNN with the BNST also an element of the extended amygdala and, hence, the hypothalamic-pituitary-adrenal axis. These regions are interconnected and function collectively to modulate socioemotional behaviors.

All ERα-ir IHC sections were visualized and imaged on a Leica TCS SPE confocal microscope using 20× objective lens with zoom factor 1.25. A set of serial image stacks (Z-stack =1 μm) was collected through the whole slice then standardized to substacks of 10 consecutive images for each ROI to control for variation in tissue thickness using the Image J software package (National Institute of Health, Bethesda, MD). The total number of ERα-ir-positive cells in each substack was then counted by hand.

All ERα RNAScope sections were imaged on a Leica TCS SPE confocal microscope using the 20× objective and a zoom factor of 1.25. As previously, a set of serial image stacks (Z-stack =1 μm) was collected through the whole slice then normalized to 7 consecutive image substacks, except for the LSV, which was substacked with 6 consecutive images. Labeling was then quantified in each image using the Image J Voxel Counter, the background subtracted, and the values averaged to give a representative density for that animal.

All measurements were made by an investigator blind to the exposure groups. For quantification of ERα-ir cell numbers and mRNA expression, section selection began at the midlevel of each ROI, then radiated bidirectionally. For each brain area, ROI and background levels were measured unilaterally from anatomically matched sections. To obtain suitable images for publication, all selected images were cropped and the brightness and contrast lightly adjusted.

In both sexes, ERα signal expression was detected in all ROIs examined, and all subregions were clearly definable. For each animal, the “sample” identified was the average of multiple consecutive sections. For ERα-ir cell counting in the AVPV, MPOA, BNSTa, and BNSTp, 3 unilateral sections were averaged, for the LSV it was 2 and for ARC, VMNvl, and MePD, it was 4. A small number of samples had fewer quality sections but were still included as detailed in Table 1.

For ERα mRNA signal, 2 unilateral sections were averaged for AVPV, MPOA, BNSTpm, BNSTpl, and LSV, whereas 4 were used for BNSTa. In BNSTp, 2 samples from the control females had fewer sections (Table 1).

### Statistical analysis

Statistical analyses were performed using GraphPad Prism, version 10 (La Jolla, CA, USA). Some samples were removed from the analysis because they were statistical outliers (defined as >2 SDs from the mean), were of poor section quality, or experienced technical issues during processing as noted for each ROI in the results. The final sample size is shown in all tables and graphs. Initial sample size was intentionally larger than needed to account for this kind of technical loss and ensure we could use only the highest quality sections. First, a planned Student *t*-test on the unexposed controls was performed to confirm detection of known and potential sex differences. Identification of known sex differences was considered validation that the study was sufficiently powered to detect biologically meaningful differences including sex-specific exposure-related effects. All data were then analyzed using 2-way ANOVA with exposure and sex as factors to identify main effects and interactions between exposure and sex, then, when indicated (by a main effect of exposure and/or significant interaction), followed up by 1-way ANOVA within sex and Fisher's least significant difference post hoc test to minimize risk of false negatives [97]. In all cases litter was the statistical unit. *P* values ≤.05 were considered statistically significant and *P* values ≤.10 were considered suggestive differences requiring replication. Data are presented as mean ± SEM.

## Results

All results are summarized in Tables 2 and 3 in all ROIs and, as anticipated, while the immunolabeled sections had robust, bright, discernable signal, the RNAScope samples had higher background and not all sections were of sufficient quality to be included in the analysis.

**Table 2:** Effects of FM 550 on ER α-ir neuron numbers in anterior hypothalamus, BNST, and LSV

Exposure/ROI (ERα-ir cell numbers)		0 µg CONTROL (CTR)	500 µg LOW	1000 µg MED	2000 µg HIGH	2-way ANOVA statistics (F, *P*)
**AVPV**	**F**	276.6 ± 104.6 (10)**	343.1 ± 43.95 (13)*^P^ ^=^ ^.06,^ ^#***P***^ **^=^** **^.09^**	381.6 ± 109.1 (10)***^,##^	277.8 ± 54.18 (8)*	**3.77, .01** **47.09, .0001** **7.08, .0003**
	**M**	183.2 ± 31.12 (14)**	264.0 ± 82.98 (10)^* *P*^ ^=^ ^.06^ ^*,###^	215.1 ± 48.67 (12)***^, # *P*^ ^=^ ^.09^	232.8 ± 16.54 (9)*^,##^	
**MPOA**	**F**	439.6 ± 106.5 (10)***	556.2 ± 83.63 (12)***^,#^	622.6 ± 197.7 (10)***^,##^	525.1 ± 87.17 (8)***	**3.28, .03** **85.59, .0001** **4.11, .009**
	**M**	309.2 ± 47.63 (14)***	382.3 ± 87.4 (10)***^,#^	305.1 ± 77.37 (11)***	320.4 ± 92.51 (12)***	
**ARC**	**F**	149.0 ± 52.17 (8)*	145.5 ± 57.14 (12)	167.6 ± 37.43 (10)	156.8 ± 22.19 (8)^* *P*^ ^=^ ^.06^	1.70, .17 *3.80, .06* 1.84, .15
	**M**	107.3 ± 32.63 (12)*	**161.3 ± 48.93 (10)** ^##^	**148.0 ± 47.04 (10)** ^#^	125.3 ± 38.44 (10)^*P^ ^=^ ^.06^	
**VMNvl**	**F**	198.5 ± 66.75 (8)*	161.2 ± 51.04 (9)	168.3 ± 42.37 (9)	**149.7 ± 39.68 (10)** ^#^	*2.52, .07* 1.25, .27 0.77, .51
	**M**	135.6 ± 52.67 (13)*	160.4 ± 43.21 (9)	**179.7 ± 40.48 (8)** ^#^	153.0 ± 35.38 (10)	
**BNSTa**	**F**	138.8 ± 25.09 (8)*	150.5 ± 47.35 (11)**	119.1 ± 38.96 (10)*	140.6 ± 42.87 (8)	0.37, .78 **17.68, .0001** **2.91, .04**
	**M**	105.5 ± 32.31 (14)*	108.1 ± 32.18 (11)**	86.53 ± 27.09 (11)*^,#*P*^ ^=^ ^.11^	121.0 ± 17.4 (10)	
**BNSTp**	**F**	384.6 ± 70.88 (9)***	386.0 ± 95.68 (10)***	341.3 ± 79.32 (11)***	293.9 ± 92.37 (8)*^,#^	1.26, .29 **64.85, .0001** **2.78, .047**
	**M**	202.9 ± 63.08 (14)***	233.9 ± 89.55 (9)***	154.6 ± 76.16 (8)***	200.6 ± 88.19 (12)*	
**LSV**	**F**	92.21 ± 41.82 (7)	67.45 ± 26.2 (10)	88.46 ± 38.64 (9)	72.31 ± 27.65 (8)	0.65, .59 1.20, .28 1.51, .22
	**M**	76.75 ± 22.01 (11)	64.78 ± 16.1 (10)	71.68 ± 13.76 (7)	78.17 ± 26.89 (9)	
**MePD**	**F**	213.7 ± 46.25 (5)*	151.8 ± 55.59 (11)^*P^ ^=^ ^.09, #*P*^ ^=^ ^.06^	216.1 ± 61.28 (10)**	194.5 ± 62.63 (8)*	0.46, .71 **25.69, .0001** **3.09, .03**
	**M**	139.7 ± 62.07 (12)*	110.6 ± 28.87 (9)^*P^ ^=^ ^.09^	137.8 ± 46.08 (8)**	129.4 ± 37.79 (9)*	

Values represent means ± SE with significant effects bolded and marginal differences underlined. The numbers in parentheses are the samples size for each experimental group. For the 2-way ANOVA statistics (F, *P*), the values from top to bottom represent the interaction, sex, and exposure.

Abbreviations: ARC, arcuate nucleus; AVPV, anteroventral periventricular nucleus; BNST, bed nucleus of the stria terminalis; BNSTa, bed nucleus of the stria terminalis, anterior part; BNSTp, bed nucleus of the stria terminalis, posterior part; BNSTpl, bed nucleus of the stria terminalis, lateral part; BNSTpm, bed nucleus of the stria terminalis, posterior medial part; ERα-ir, estrogen receptor α immunoreactive; F, female; FM 550, Firemaster 550; LSV, ventral part of the lateral septum; M, male.

Significant sex differences are shown as *P ≤ .05, **P ≤ .01, and ***P ≤ .001.

Significant effects of exposure compared to controls are shown as #*P* ≤ .05, ##*P* ≤ .01, and ###*P* ≤ .001.

*P* ≤ .10 represents a marginal difference.

**Table 3:** Effect of FM 550 exposure on sexually dimorphic ERα-ir neuron numbers/ERα mRNA levels in hypothalamus, amygdala, BNST and LSV

ROI/exposure (ERα-ir)	0 µg CONTROL	500 µg LOW	1000 µg MED	2000 µg HIGH	0 µg CONTROL (mRNA)	2000 µg HIGH (mRNA)
**AVPV**	**F** **>** **M**	**↑** F = M **⇑**	**⇑ F** **>** **M ↑**	**F** **>** **M ⇑**	**F** **>** **M**	**⇑ F** **>** **M**
**MPOA**	**F** **>** **M**	**⇑ F** **>** **M ⇑**	**⇑ F** **>** **M**	**F** **>** **M**	**F** **>** **M**	**F** **>** **M**
**ARC**	**F** **>** **M**	F = M **⇑**	F = M **⇑**	F = M	N/A	N/A
**VMNvl**	**F** **>** **M**	F = M	F = M **⇑**	**⇓** F = M	N/A	N/A
**MePD**	**F** **>** **M**	↓ F = M	**F** **>** **M**	**F** **>** **M**	N/A	N/A
**LSV**	F = M	F = M	F = M	F = M	F = M	F = M
**BNSTa**	**F** **>** **M**	**F** **>** **M**	**F** **>** **M**	F = M	F = M	F = M
**BNSTp**	**F** **>** **M**	**F** **>** **M**	**F** **>** **M**	**⇓ F** **>** **M**	**BNSTpm** F = M **BNSTpl** F = M	F = M **⇑** F = M

All observed sex differences are bolded. For some groups, FM 550 exposure disrupted sex differences seen in unexposed controls. Those effects are underlined with **⇑, ⇓** representing the direction in which the signal in each sex changed (increased or decreased) compared with same sex controls. Marginal changes in signal are represented by ↓, ↑ (increased or decreased). F = M represents no sex difference, F > M represents females have more ERα-ir cells number or higher mRNA expression levels than males.

Abbreviations: ARC, arcuate nucleus; AVPV, anteroventral periventricular nucleus; BNST, bed nucleus of the stria terminalis; BNSTa, bed nucleus of the stria terminalis, anterior part; BNSTp, bed nucleus of the stria terminalis, posterior part; BNSTpl, bed nucleus of the stria terminalis, lateral part; BNSTpm, bed nucleus of the stria terminalis, posterior medial part; ERα-ir, estrogen receptor α immunoreactive; F, female; FM 550, Firemaster 550; LSV, ventral part of the lateral septum; M, male.

### AVPV

ERα expression in the prairie vole AVPV was sexually dimorphic. Expression level was affected by FM 550 exposure, sex and dose dependently.

### AVPV ERα-ir cell numbers

**Figure 2:**
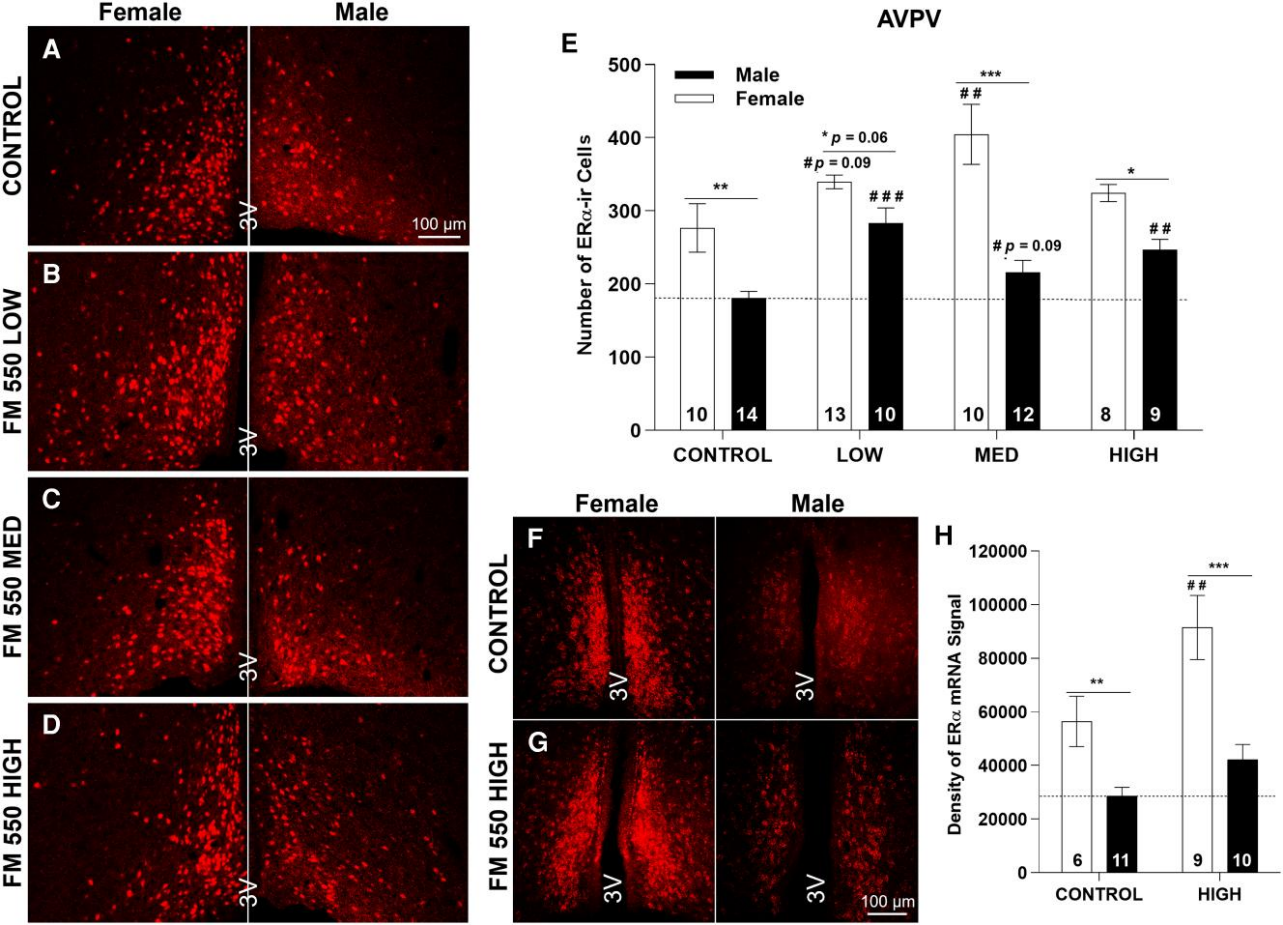
Representative images from each exposure group for ERα-ir (A to D) or ERα mRNA (F and G). Numbers in each column of E and H represent the sample size in each exposure group. *P ≤ .05, **P ≤ .01, ***P ≤ .001 represent significant differences between sexes. ^##^*P* ≤ .01, ^###^*P* ≤ .001 represent significant effects of exposure within each sex. Marginal differences (*P* ≤ .10) are also shown. Bars represent mean ± SE.

Intense ERα-ir was detected in the AVPV of both males and females regardless of exposure group (Fig. 2A to 2D, Table 2). Two-way ANOVA indicated a significant effect of sex (F(1,78) = 47.09, *P* ≤ .001) and exposure (F(3,78) = 7.08, *P* ≤ .001) plus a significant interaction (F(3,78) = 3.77, *P* ≤ .01; Table 2). Follow-up 1-way ANOVA within sex revealed a significant effect of exposure in females (F(3,37) = 3.77, *P* ≤ .01) and males (F(3,41) = 8.48, *P* ≤ .01; Fig. 2E). In females, numbers were higher in the MED (*P* ≤ .01) and marginally higher in the LOW (*P* = .09) groups. In males, AVPV ERα-ir cell numbers were significantly increased (Fig. 2A to 2D right panels, and E) in the LOW (*P* ≤ .001) and HIGH groups (*P* ≤ .01), with a marginal increase in the MED group (*P* = .09; Fig. 2E).

As expected, a sex difference in AVPV ERα-ir cell numbers was observed in the control groups, with females having more (*P* ≤ .01). Exposure did not eliminate this difference, even if ERα-ir cell numbers were significantly altered in 1 sex, although it came close in the LOW group (*P* = .06; (Fig. 2A to 2E, Table 2).

### AVPV ERα mRNA expression

For the RNAScope analysis, 2-way ANOVA revealed a significant effect of sex (F(1,32) = 23.88, *P* ≤ .001) and exposure (F(1, 32) = 9.56, *P* ≤ .01) but no interaction (F(1,32) = 1.88, *P* = .18) (Fig. 2F to 2H, and Table 3). The anticipated sex difference in ERα mRNA expression was observed in the control groups with expression higher in females, and this pattern was not changed by high-dose exposure (Fig. 2H and Table 3)

### MPOA

ERα expression in the MPOA was more intense than in the AVPV, sexually dimorphic, and affected by FM 550, mainly in females.

### MPOA ERα-ir cell numbers

**Figure 3:**
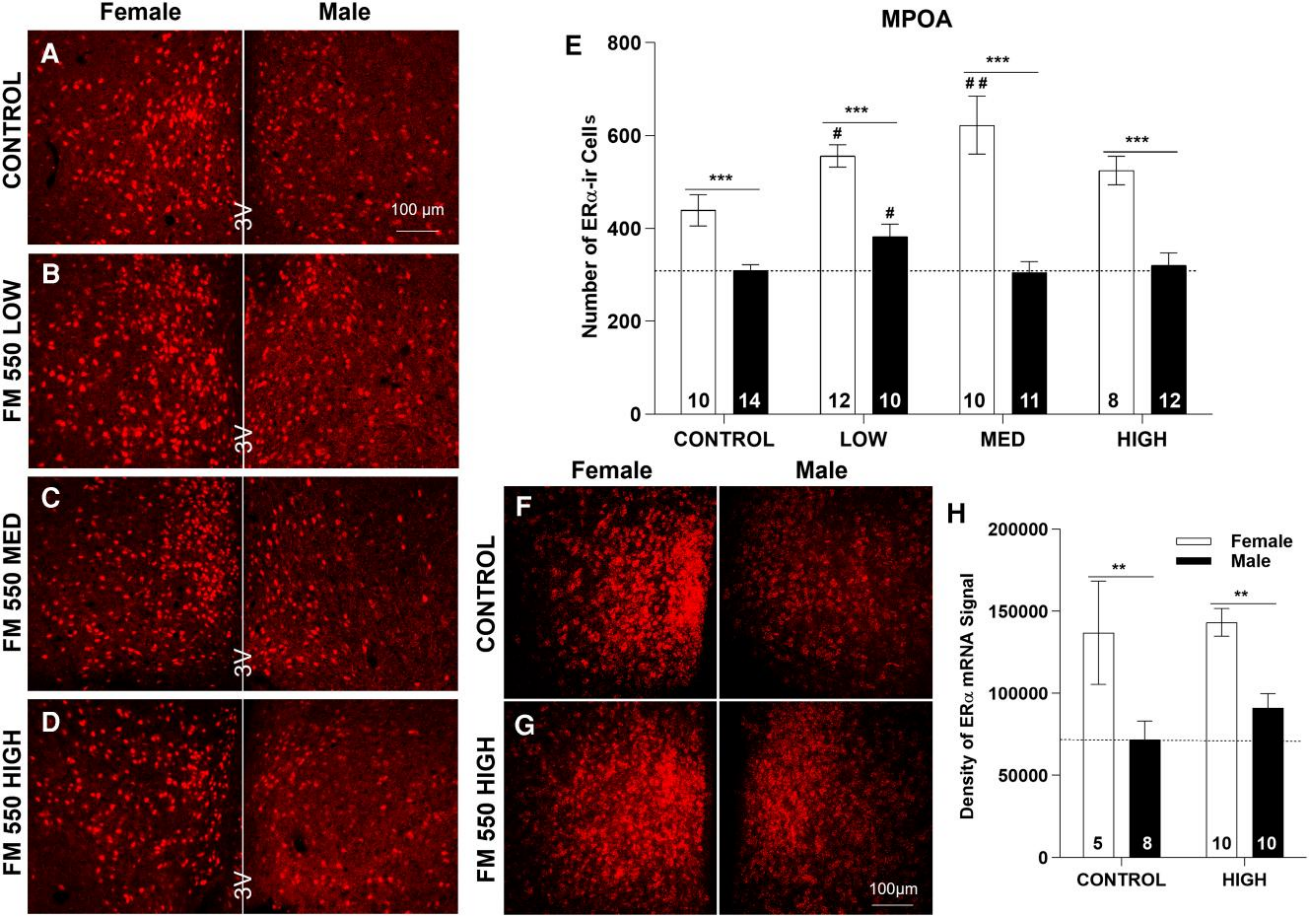
Representative images from each exposure group for ERα-ir (A-D) or ERα mRNA (F and G). Numbers in each column of E and H represent the sample size in each exposure group. **P ≤ .01, ***P ≤ .001 represent significant difference between sexes. ^#^*P* ≤ .05, ^##^*P* ≤ .01 represent significant effects of exposure within each sex. Bars represent mean ± SE.

Two-way ANOVA revealed a significant effect of sex (F(1,79) = 85.59, *P* ≤ .001), exposure (F(3,79) = 4.11, *P* ≤ .01), and an interaction (F(3,79) = 3.28, *P* ≤ .05; Table 2). Follow-up 1-way ANOVA within sex revealed an effect of exposure in females (F(3,36) = 3.57, *P* ≤ .05), but only a marginal effect of exposure in males (F(3,43) = 2.32, *P* = .09; Fig. 3A to 3E). In females, both low and medium doses of FM 550 significantly increased MPOA ERα-ir cell numbers (*P* ≤ .05 and *P* ≤ .01), but not the high dose (Fig. 3A to 3E). In males, MPOA ERα-ir cell numbers were only increased in the low-dose-exposure group (*P* ≤ .05; Fig. 3A to 3E) but not MED or HIGH. Control females had more ERα-ir cells than males (*P* ≤ .001) as expected, and this sexually dimorphic pattern was not affected by any dose of FM 550 (Fig. 3E).

### MPOA ERα mRNA expression

Two-way ANOVA revealed a significant effect of sex (F(1,29) = 19.04, *P* ≤ .001), but no effect of exposure (F(1, 29) = 0.90, *P* = .35), or an interaction (F(1,29) = 0.23, *P* = .64). The sex difference in mRNA levels was found in both CONTROL and HIGH groups, with expression higher in females than males (Fig. 3F and 3H and Table 3).

### ARC

**Figure 4:**
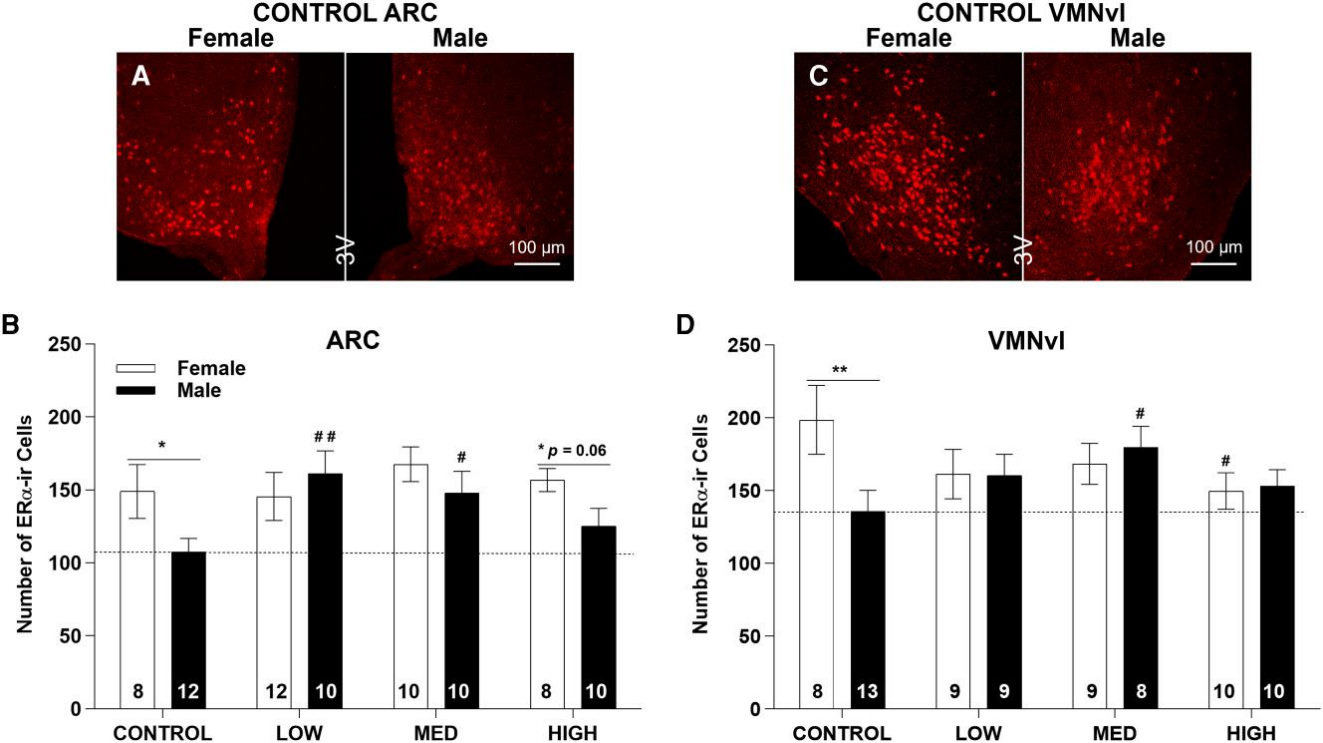
Representative images of ERα-ir in the control ARC (A) and VMNvl (C). Numbers in each column of (B and D) represent the sample size in each exposure group. *P ≤ .05, **P ≤ .01 represent significant difference between sexes. ^#^*P* ≤ .05, ^##^*P* ≤ .01 represent significant difference of exposure within each sex. The nearly significant sex difference in the HIGH group (*P* ≤ .10) is also depicted. Bars represent mean ± SE.

ERα-ir signal was readily detectable in the ARC (Fig. 4A). Two-way ANOVA revealed a marginal effect of sex (F(1,72) = 3.80, *P* = .06), no effect of exposure (F(3, 72) = 1.84, *P* = .15) and no interaction (F(3, 72) = 1.70, *P* = .17). A sex difference in ERα-ir cell numbers was observed in the CONTROL group (*P* ≤ .05) with higher numbers in females. This sex difference was eliminated in the LOW and MED groups (Fig. 4B) and became marginal in the HIGH group with *P* = .06 (Fig. 4B). To gain further information for overall data interpretation, in males, an unprotected post hoc 1-way ANOVA (F(3, 38) = 3.57, *P* ≤ .05) was run and identified increased ERα-ir cell numbers in the LOW (*P* ≤ .01) and MED *P* ≤ .05 groups (Fig. 4B). This result should be evaluated with caution given it was an unplanned comparison.

### VMNvl

ERα-ir signal was mainly confined to the VMNvl, as expected. Two-way ANOVA revealed no significant effect of sex (F(1, 68) = 1.25, *P* = .27) or exposure (F(3, 68) = 0.77, *P* = .51) and only a marginal interaction (F(3, 68) = 2.52, *P* = .07). As expected, density was sexually dimorphic in the CONTROL group with females having more ERα-ir cells than males. This sex difference was absent in all exposure groups (Fig. 4C and 4D and Tables 1 and 2). Of note, unprotected post hoc *t*-tests revealed significantly (*P* ≤ .05) decreased ERα-ir cell numbers in HIGH female VMNvl, and increased numbers in MED male VMNvl, but these observations should be evaluated with caution given it was an unplanned comparison.

### BNSTa

The BNST is a complex structure comprising multiple subnuclei. We focused on the mid-level sections in each ROI to assure sampling consistency. ERα signal was nuclear in both BNSTa and BNSTp and readily observable with stronger signal in BNSTp than in BNSTa.

### BNSTa ERα-ir cell numbers

**Figure 5:**
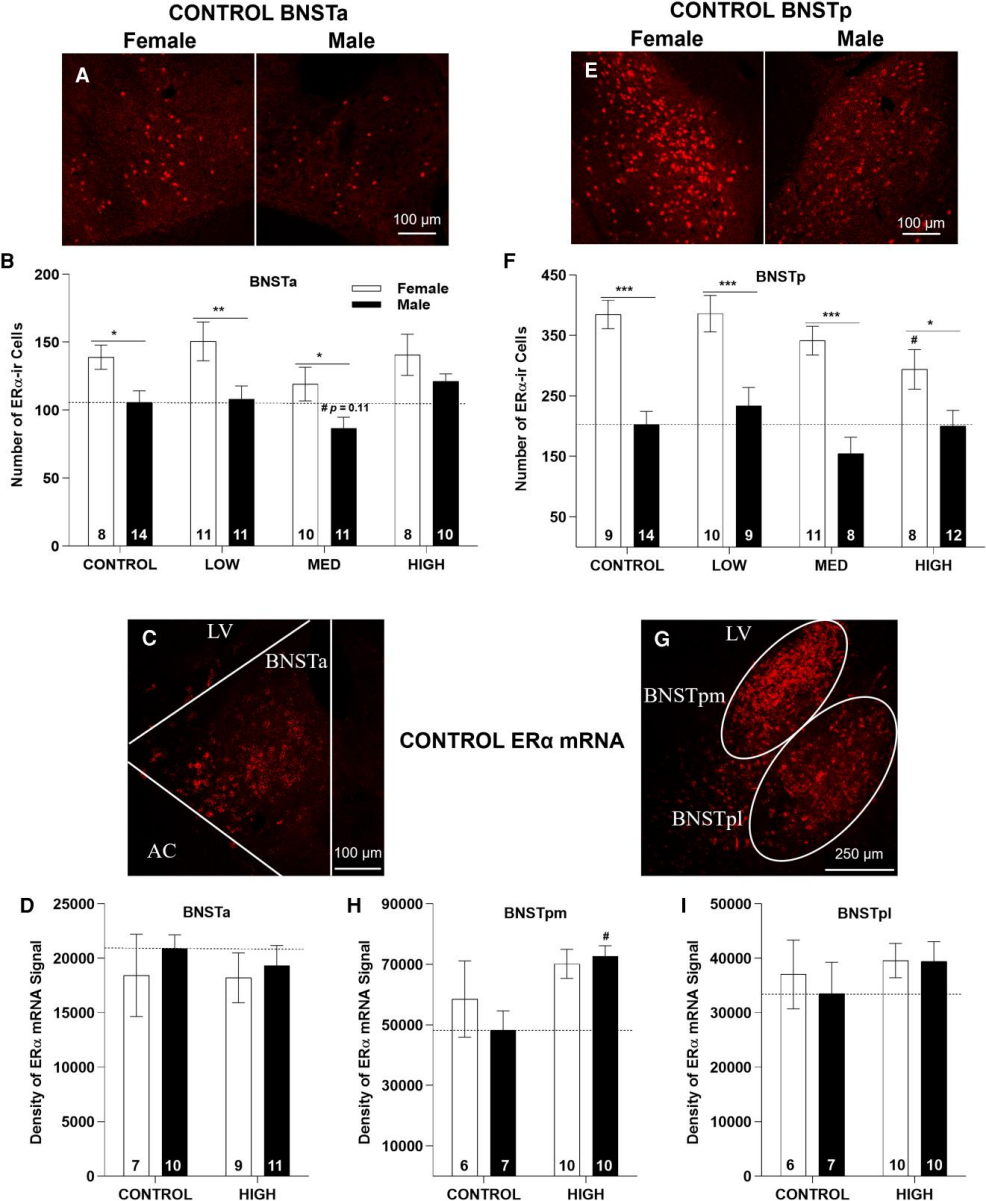
Representative images of ERα-ir (A and E) or ERα mRNA (C and G). Numbers in each column of all graphs represent the sample size in each exposure group. *P ≤ .05, **P ≤ .01, ***P ≤ .001 represent significant difference between sexes. ^#^*P* ≤ .05 represents significant effects of exposure within each sex. Bars represent mean ± SE.

Two-way ANOVA revealed a significant effect of sex (F(1,75) = 17.68, *P* ≤ .001) and exposure (F(3,75) = 2.91, *P* ≤ .05), but no interaction (F(3,75) = 0.37, *P* = .78; Fig. 5A and 5B and Table 2). Follow-up 1-way ANOVA found no significant effect of exposure in females (F(3, 33) = 1.10, *P* = .36) and only a marginal effect in males (F(3, 42) = 2.65, *P* = .06; Fig. 5B and Table 2). In MED males, BNSTa ERα-ir cell numbers were marginally higher but not statistically significant (*P* = .11). In CONTROLs, BNSTa ERα-ir cell numbers were sexually dimorphic, with more in females. This difference was not present in the HIGH group (*P* = .23; Fig. 5A and 5B and Table 2).

### BNSTa ERα mRNA expression

Two-way ANOVA revealed no effect of sex (F(1,33) = 0.64, *P* = .43), exposure (F(1, 33) = 0.16, *P* = .69), nor an interaction (F(1,33) = 0.09, *P* = .76; Fig. 5C and 5D). No sex differences were identified in either group (Tables 2 and 3).

### BNSTp

#### BNSTp ERα-ir cell numbers

Two-way ANOVA revealed a significant effect of sex (F(1,73) = 64.85, *P* ≤ .001) and exposure (F(3,73) = 2.78, *P* ≤ .05), but not interaction (F(3,73) = 1.26, *P* = .29; Table 2). In females, 1-way ANOVA detected a marginal exposure effect (F(3,34) = 2.28, *P* = .097) with levels lower only in the HIGH group (*P* ≤ .05). Levels were unaffected in males (F(3, 39) = 1.28, *P* = .29; Table 2). Female CONTROLs had more ERα-ir cells than males (*P* ≤ .001) and exposure did not affect this sex difference in any dose group (Fig. 5E and 5F, Tables 2 and 3).

#### BNSTp ERα mRNA expression

ERα mRNA signal in the BNSTp was notably denser than in the BNSTa (Fig. 5G). The mRNA signal in the BNSTp was observed as clusters that could be distinguished as the BNST postmedial portion (BNSTpm) and BNST postlateral portion (BNSTpl) (Figs. 1D and 5G and Table 2). Two-way ANOVA detected a significant effect of exposure (F(1, 29) = 7.66, *P* ≤ .01) in BNSTpm, with levels higher in exposed males. There was no effect of sex (F(1, 29) = 0.36, *P* = .55) nor an interaction (F(1, 29) = 0.97, *P* = .33). In BNSTpl, no effects of (sex F(1, 29) = 0.17, *P* = .69), exposure (F(1, 29) = 0.89, *P* = .35), nor an interaction (F(1, 29) = 0.14, *P* = .71) was found. No sex differences were found in either group (Fig. 5H and 5I, Tables 2 and 3).

### LSV

ERα signal detected in the LSV was not as high as in the other ROIs, but still quantifiable in most available sections.

### LSV ERα-ir cell numbers

**Figure 6:**
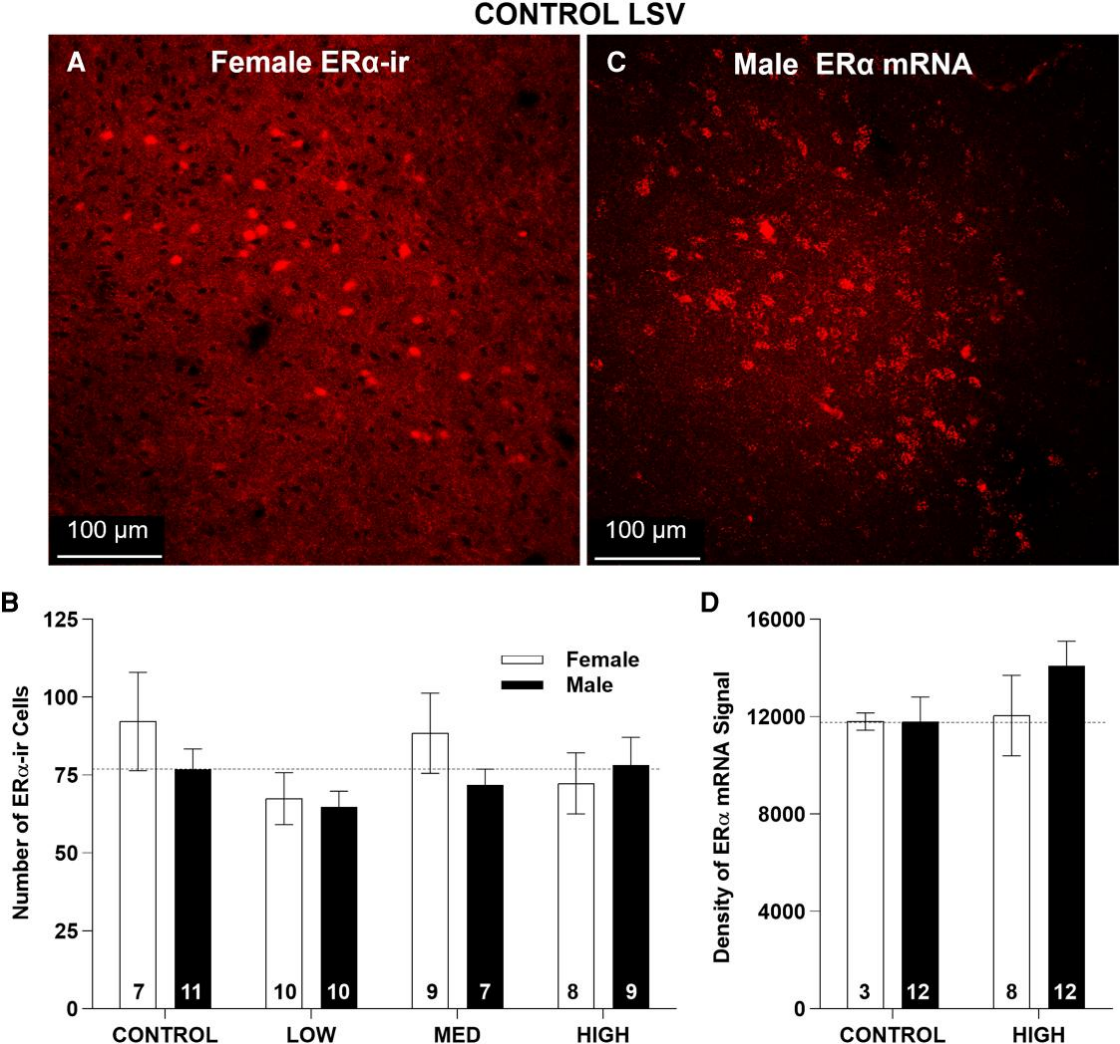
Representative images of LSV ERα-ir in a control female (A) and ERα mRNA in a control male (B). Numbers in each column of (C and D) represent the sample size in each exposure group. No effect of sex or exposure was found for this region. Bars represent mean ± SE.

Two-way ANOVA revealed no effect of sex (F(1,63) = 1.20, *P* = .28), exposure (F(3, 63) = 1.51, *P* = .22) or interaction (F(3,63) = 0.65, *P* = .59; Table 2, Fig. 6A and 6B). Density was not sexually dimorphic in any group (Tables 2 and 3, Fig. 6B).

### LSV ERα mRNA expression

ERα mRNA levels did not differ by sex (F(1, 31) = 0.48, *P* = .49) or exposure (F(1, 31) = 0.74, *P* = .40) and there was no interaction (F(1, 31) = 0.49, *P* = .49). mRNA expression was not sexually dimorphic in either group (Fig. 6C and 6D, and Tables 2 and 3).

### MePD

**Figure 7:**
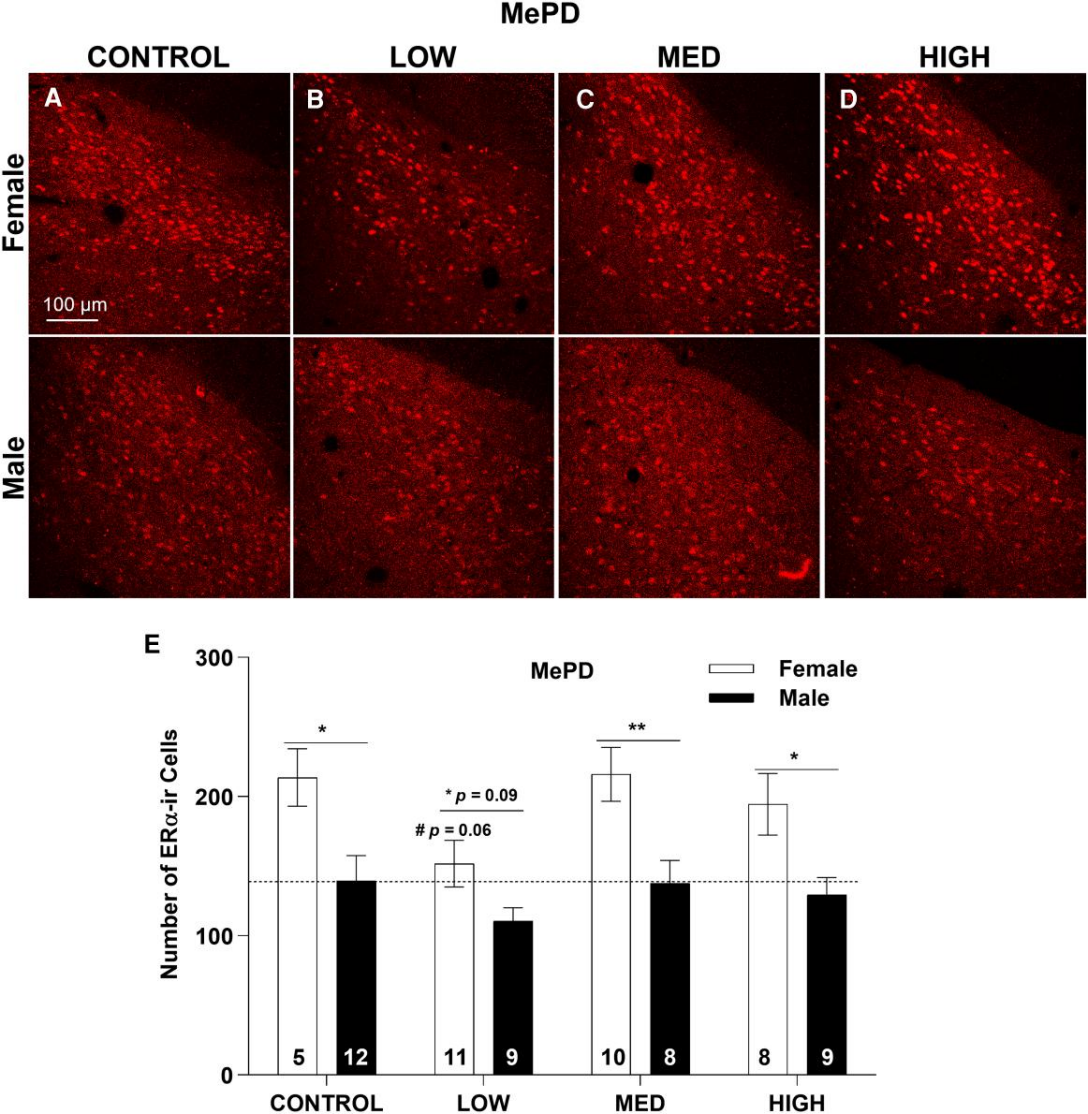
Representative images of ERα-ir in control and FM 550 exposure groups (A-D). Numbers in each column of (E) represent the sample size in each exposure group. *P ≤ .05, **P ≤ .01 represent significant difference between sexes. *P = .09 represents marginal sex difference in low-dose exposure groups, ^#^*P* ≤ .06 represents marginal difference in low dose female group. Bars represent mean ± SE.

Immunolabeling of ERα in the MePD was strong and readily detectable. Two-way ANOVA identified a significant effect of sex (F(1, 64) = 25.69, *P* ≤ .001) and exposure (F(3, 64) = 3.09, *P* ≤ .05), but no significant interaction (F(3, 64) = 0.46, *P* = .71; Fig. 7 and Table 2). Follow-up 1-way ANOVA revealed no effect of exposure in males (F(3, 34) = 0.75, *P* = .53), and only a marginal effect of exposure in females (F(3, 30) = 2.57, *P* = .07) with fewer ERα-ir cell numbers in the LOW group (*P* = .06; Fig. 7E). As, expected CONTROL females had more ERα-ir cells than males (*P* ≤ .05; Fig. 7A and 7E, Tables 2 and 3). Low-dose FM 550 exposure eliminated this sex difference, largely driven by decreased ERα-ir cell number in females, but not MED or HIGH (Fig. 7E, Tables 2 and 3).

## Discussion

As anticipated, FM 550 impacts on SBNN ERα were sex and region specific. In males, the most consistent effects were increased ERα-ir cell numbers in AVPV, MPOA, and (to some degree) ARC whereas in females, upregulation was observed in AVPV and MPOA with suggestive evidence of downregulation in VMNvl and MePD. Although immunolabeling was robust, mRNA signal was less definitive likely because fixed tissue is not optimal for RNAscope. The present findings align with extensive prior work suggesting that ERα is a hub target for EDCs, particularly in the AVPV/MPOA [50, 51, 58, 98, 99]. Also consistent with prior work, exposure often abrogated or eliminated sex differences even in cases where a main effect of exposure was not detected, particularly in the LSV, VMNvl, and ARC. Notably, FM550 exposure tended to increase ERα-ir cell numbers in the anterior hypothalamus regardless of sex, but decrease them in the female posterior hypothalamus, amygdala, and extended amygdala; a pattern meriting further exploration using a systems-level approach. Impacted ERα neuronal populations express or are influenced by neuropeptides, neurosteroids, and other signaling molecules that govern prosocial behaviors, which is the ongoing direction of this work, but also reproductive and metabolic functions; systems that have also been shown to be altered by developmental FR exposure [40, 100-103]. Collectively, these data add to growing evidence that FM 550 FRs are neuroendocrine disruptors that can induce persistent impacts across the developing brain.

Significantly, this study advances experimental approaches for understanding how environmental exposures may be contributing to rising rates of ASD and other NDDs by using a particularly human-relevant rodent species for the specific phenotype of interest, with sex as a biological variable, and an National Institute of Mental Health-derived framework for linking behavioral traits to their biological drivers. Hence, these studies serve as a model for how “fit for purpose” in vivo studies could be conducted and used under the 3R framework for toxicological assessments seeking to link chemical exposures to NDD-related behaviors.

These data are also significant because FRs are among the most common EDCs and extensively found in a wide range of products including electronics, building materials, foam-based furniture, and infant products [104, 105]. FRs readily leach into the environment and accumulate in home or workplace dust that is subsequently ingested or inhaled [17, 89, 106-109]. Hence, they are prevalent global pollutants, often with long half-lives [110, 111]. Legacy brominated FRs are known thyroid disruptors [112], consequently, “next-generation” FRs, including newer brominated forms and OPEs found in FM 550, were developed to avoid this mode of action. Unfortunately, they have been shown to have other modes of endocrine disruption and DNT [11, 113]. Because OPEs are more rapidly metabolized and excreted than most BFRs [114], it has been asserted that they are a safer alternative to BFRs; but compounding evidence from us and others indicate they can disrupt monoamine function and key aspects of neurodevelopment including axon guidance [11, 12, 59, 115, 116]. The data generated herein add to this growing literature by identifying that the selective disruption of ERα expression in regions of the SBNN may be a key mode of action.

### Anterior hypothalamus

In the AVPV, FM 550 exposure increased ERα-ir cell numbers in both sexes, with a nonmonotonic dose response in females. The same pattern was also observed in the female MPOA. Notably, although AVPV mRNA levels were elevated in the HIGH females, cell density was unaffected, suggesting transcriptional changes may not consistently signify protein-level changes. Interpretation must be made with caution, however, because of technical limitations experienced using RNAscope in fixed tissue.

It is well established that the AVPV and MPOA have some of the most pronounced sex differences in ERα density and expression in multiple rodent species [85], including the prairie vole [117-120], with levels higher in females than males. Those distinctive sex differences were identified in the control animals, demonstrating sufficient statistical power was achieved to detect biologically meaningful differences in these key regulators of female reproductive behavior and physiology. We and others have previously shown that multiple aspects of AVPV and MPOA structure, composition, and function, in addition to ERα-ir density and expression, are vulnerable to EDCs, resulting in impaired sex behavior and reproductive function, particularly in females [50, 56, 58, 121-123]. Hence, the FM 550 effects observed herein are consistent with compounding evidence that the full mixture and its OPFR components are endocrine disrupting [103, 124].

Long known to be essential for female and male sexual behavior [125-128], the MPOA is an integrative area for mesolimbic circuits and, consequently, motivated socioemotional and related behaviors [129, 130]. It also plays a fundamental regulatory role in maternal behavior [131-133] and is a downstream target of ERα neurons in the BNST gating mating and related aggressive behaviors in males [133]. Knockdown of ERα or blockade of estrogen in the MPOA disrupts both female sex behavior [134] and maternal behavior [135-138]. Observed higher levels in FR-exposed females suggests maternal behavior is likely not impaired, an interpretation consistent with our prior observation that female partner attachment remains intact if not heightened [45, 47, 49]. Impacts on parental care, mating behavior, and aggression, however, remain to be determined.

### BNST and MePD

Given their fundamental roles in male affiliation and pair bonding, both of which we have repeatedly observed to be compromised by developmental FM 550 exposure [45, 47, 49], we postulated the BNST and MePD ERα populations could be particularly affected. For example, it has previously been shown in male prairie and pine voles as well as Siberian hamsters that ERα levels in BNSTp and MePD, are inversely associated with high prosociality including pair bond formation and alloparental care [119, 139-141]. ERα overexpression by viral vector in MePD or BNSTp of male prairie voles inhibits or disrupts partner preferences and alloparental behavior [141, 142]. Males in polygynous species, such as meadow voles, montane voles, and dwarf hamsters, express markedly higher ERα levels [119, 139, 140]. Hence, we anticipated exposure would elevate ERα, but ultimately found little evidence to support that hypothesis. Because prior work has shown that the female BNSTp contains more ERα-ir cells than males in both adult and weanling prairie voles [118-120], an observation recapitulated here, for both the BNSTp and BNSTa, insufficient statistical power is not likely a factor for the lack of observed effects. Some evidence of abrogation or loss of well-characterized sex differences was found; the functional consequences of which, if any, remain to be determined.

It is possible targets other than ERα may be more significantly impacted in these regions, particularly AVP and its receptor. Both the BNSTp and MePD have steroid-sensitive AVP cell populations [143] that profoundly regulate prosocial traits including attachment [144]. BNST AVP mRNA and cell numbers increase in male prairie voles following 72 hours of pairing and promote pair bonding, a phenomenon not seen in the promiscuous meadow vole [145]. Classic work has well-established that AVP action in the BNST and related SBNN nuclei promote male affiliative and prosocial behavior, including pair bonding [145-147]. In rats, AVP-ir cells in the BNST and MeA coexpress ERα, along with AR and PR. Steroids act directly on these cells to modify behavior [144]; hence, FRs and other EDCs may act similarly even if ERα expression itself is unaffected.

Additionally, although no effects on ERα were identified here, the LSV is a related region that remains an ROI for future study because it is also is involved in emotional behaviors including anxiety regulation, fear, and defensive/aggressive behaviors [148]. The LSV expresses OXTR, ERβ, and GPER, all of which may be vulnerable to FR exposure. Ongoing work is focusing on these other potential targets. AVP and oxytocin are of particular interest because these nonapeptides are known to profoundly influence affiliation and attachment in all monogamous mammals examined to date, including humans [70, 72, 74].

Dopaminergic neurons are also present in the prairie vole MePD and BNSTp [149] and gate attachment along with other socioemotional traits [119, 139, 149], implicating the mesolimbic dopamine system as an additional endpoint of interest for future investigation. Surprisingly little EDC work has focused on this pathway [98, 122]; most neurotoxicity work (including with NAMs) has focused on nigrostriatal dopamine [150]. There are suggestions, however, that it could be vulnerable to FRs and FM 550 specifically [115, 151]. Our laboratory has shown in a related set of animals, for example, that FM 550-exposed females have more A13 TH neurons in the zona incerta than controls with more sporadic effects in males [49]. We have also reported evidence of disrupted fetal DA system development in prenatally OPFR-exposed rats [115].

### Mediobasal hypothalamus

In the control animals, here we found ARC ERα cell density to be higher in females; an observation that differs from prior work reporting no sex difference in adults [118, 119]. Hence, the observed outcomes in the ARC should be interpreted with caution. The reason for this difference is unclear but could be related to anatomical differences in the subsections quantified or antigenic differences between the antibodies used across studies. Notably, in weanlings, a directionally consistent sex difference has been reported [120], suggesting the possibility that it lingers in our colony and diminishes at a greater age. In the prairie vole VMNvl, ERα expression is sexually dimorphic neonatally through adulthood, with consistently higher levels in females and this was recapitulated herein [117-119, 149]. Other rodents, such as rats [95, 152, 153], have similar sex differences.

These sex differences are of interest because the most consistently observed effect of FM 550 in the ARC and VMN was the elimination of prominent sex differences in ERα-ir cell numbers, apparently because of exposure-related changes in males. Elimination of sex differences is a common outcome for EDCs even if effects in each sex do not reach statistical significance [98, 98, 154-156]. Impacts on socioemotional behavior and related physiology remain unclear but potentially consequential given that nearly all NDDs and mental health disorders have a sex-bias in both incidence and clinical presentation with steroid hormones, particularly estrogen, hypothesize to play a central role [157-161].

Although subtle and requiring replication, the observed outcomes may be more related to metabolic function than sociality and corroborate reported OPFR-related homeostatic effects in other rodent species. Using Wistar rats, our group was the first to show that FM 550 acts as an obesogen in vivo, with developmental exposure producing weight gain from PND 21 to PND 220, most notably in males, along with thickening of the lateral ventricle, and evidence of glucose intolerance on PND 120 in females, at an oral dose of 1000 μg/day [36]. It has long been known that in the ARC/VMN, estrogens, and ERα specifically, play fundamental roles in the neuroendocrine control of metabolic function, including body weight maintenance, across the lifespan via complex neuroendocrine systems that intersect with reproductive and other hedonic pathways [162-167]. Work dating back decades has shown that the ARC in particular contains a diversity of neuronal subpopulations (reviewed in [168]), that selectively coexpress ERα and respond to endogenous estrogens for the regulation of metabolism, food intake, energy balance, and reproduction [85, 85, 167-172].

Primarily using mice, but also myriad in vitro systems, other groups have shown that maternal exposure to OPFRs, including TPHP, exacerbates body weight gain and adiposity, particularly when fed a high-fat diet, and decreases glucose clearance [173-177]. Detailed work suggests the mode of action involves ERα but also a significant role for PPARγ [34, 176], along with other neuropeptides, with marked sex differences in vulnerability [103, 178-180].

## Summary and conclusions

The present studies build on our prior work in Wistar rats and prairie voles showing that developmental exposure to the commercial FR mixture FM 550, or its component classes (BFRs or OPFRs) elevates anxiety-like behaviors in females and decreases aspects of sociality in both sexes, most notably pair bonding in males [33, 46, 47] by working toward identifying mode(s) of action. The results implicate the anterior hypothalamus as a particularly vulnerable target but also corroborate to some degree prior work in mice and other model systems revealing ERα-related effects of developmental OPFR exposure on metabolic function and adiposity.

Limitations include suboptimal signal from the RNAscope labeling and detection of a possible sex difference in ARC ERα expression where, in previous publications, none was observed. Results of unprotected statistical tests (ARC and VMN data) should be interpreted with caution and merit further investigation. Advantages include use of a more human-relevant animal model for socioemotional traits than traditional laboratory rats or mice, and an experimental framework specifically designed to probe the biological basis of human behavioral traits including attachments.

Clinically, NDDs are defined behaviorally and lack defining pathology, making experimentally causality challenging for toxicologists because they are used to relying on pathology data from prescriptive guideline studies, mostly in rats. Yet, those protocols lack socioemotional endpoints and do not examine the neuroendocrine systems that underlie these behaviors [3, 181, 182]. As toxicologists move to embrace NAMs, alternative animals with greater translational relevance for the outcome of interest, such as prairie voles and attachment [76], merit greater consideration. NAMs covering the SBNN, including the mesolimbic dopamine system, are also lacking.

As anticipated, exposure-related effects were dependent on sex, dose, and region, emphasizing the importance of considering sex as a biological variable. Our data provide support that disruption of ERα expression in the SBNN may be a mechanism underlying disruption of socioemotional behavior, energy balance, and, potentially parental behaviors and aggression. Future work will examine related neuroendocrine receptor targets other than ERα, such as ERβ, GPER, neuropeptides, neurotransmitters, and metabolic enzymes.

## Data Availability

Some or all datasets generated during and/or analyzed during the current study are not publicly available but are available from the corresponding author on reasonable request.

## Abbreviations

ARC: arcuate nucleus
ASD: autism spectrum disorder
AVPV: anteroventral periventricular nucleus
BFR: brominated flame retardant
BNST: bed nucleus of the stria terminalis
BW: body weight
DNT: developmental neurotoxicant
EDC: endocrine disrupting chemical
ERα: estrogen receptor alpha
ERα-ir: estrogen receptor alpha immunoreactive
FM 550: Firemaster 550
FR: flame retardant
GPER: G protein-coupled estrogen receptor 1
IHC: immunohistochemistry
KPBS: potassium phosphate buffer solution
MeA: nuclear groups within the medial and extended amygdala
MPOA: medial preoptic area
NAM: new approach methodology
NDD: neurodevelopmental disorder
OPE: organophosphate ester
PND: postnatal day
RDoC: Research Domain Criteria
ROI: region of interest
RT: room temperature
SBNN: social brain neural network
TPHP: triphenyl phosphate
VMN: ventromedial nucleus
